# Global expression analysis of the brown alga *Ectocarpus siliculosus *(Phaeophyceae) reveals large-scale reprogramming of the transcriptome in response to abiotic stress

**DOI:** 10.1186/gb-2009-10-6-r66

**Published:** 2009-06-16

**Authors:** Simon M Dittami, Delphine Scornet, Jean-Louis Petit, Béatrice Ségurens, Corinne Da Silva, Erwan Corre, Michael Dondrup, Karl-Heinz Glatting, Rainer König, Lieven Sterck, Pierre Rouzé, Yves Van de Peer, J Mark Cock, Catherine Boyen, Thierry Tonon

**Affiliations:** 1 UPMC Univ Paris 6, UMR 7139 Végétaux marins et Biomolécules, Station Biologique, 29680 Roscoff, France; 2 CNRS, UMR 7139 Végétaux marins et Biomolécules, Station Biologique, 29680 Roscoff, France; 3 CEA, DSV, Institut de Génomique, Génoscope, rue Gaston Crémieux, CP5706, 91057 Evry, France; 4 CNRS, UMR 8030 Génomique métabolique des genomes, rue Gaston Crémieux, CP5706, 91057 Evry, France; 5 Université d'Evry, UMR 8030 Génomique métabolique des genomes, 91057 Evry, France; 6 SIG-FR 2424 CNRS UPMC, Station Biologique, 29680 Roscoff, France; 7 Center for Biotechnology (CeBiTec), University of Bielefeld, 33594 Bielefeld, Germany; 8 German Cancer Research Center (DKFZ), Im Neuenheimer Feld 580, 69120 Heidelberg, Germany; 9 VIB Department of Plant Systems Biology, Ghent University, 9052 Ghent, Belgium

## Abstract

**Background:**

Brown algae (Phaeophyceae) are phylogenetically distant from red and green algae and an important component of the coastal ecosystem. They have developed unique mechanisms that allow them to inhabit the intertidal zone, an environment with high levels of abiotic stress. *Ectocarpus siliculosus *is being established as a genetic and genomic model for the brown algal lineage, but little is known about its response to abiotic stress.

**Results:**

Here we examine the transcriptomic changes that occur during the short-term acclimation of *E. siliculosus *to three different abiotic stress conditions (hyposaline, hypersaline and oxidative stress). Our results show that almost 70% of the expressed genes are regulated in response to at least one of these stressors. Although there are several common elements with terrestrial plants, such as repression of growth-related genes, switching from primary production to protein and nutrient recycling processes, and induction of genes involved in vesicular trafficking, many of the stress-regulated genes are either not known to respond to stress in other organisms or are have been found exclusively in *E. siliculosus*.

**Conclusions:**

This first large-scale transcriptomic study of a brown alga demonstrates that, unlike terrestrial plants, *E. siliculosus *undergoes extensive reprogramming of its transcriptome during the acclimation to mild abiotic stress. We identify several new genes and pathways with a putative function in the stress response and thus pave the way for more detailed investigations of the mechanisms underlying the stress tolerance ofbrown algae.

## Abstract 2

The brown alga *Ectocarpus siliculosus*, unlike terrestrial plants, undergoes extensive reprogramming of its transcriptome during the acclimation to mild abiotic stress.

## Background

The brown algae (Phaeophyceae) are photosynthetic organisms, derived from a secondary endosymbiosis [1], that have evolved complex multicellularity independently of other major groups such as animals, green plants, fungi, and red algae. They belong to the heterokont lineage, together with diatoms and oomycetes, and are hence very distant phylogenetically, not only from land plants, animals, and fungi, but also from red and green algae [2]. Many brown algae inhabit the intertidal zone, an environment of rapidly changing physical conditions due to the turning tides. Others form kelp forests in cold and temperate waters as well as in deep-waters of tropical regions [3,4]. Brown algae, in terms of biomass, are the primary organisms in such ecosystems and, as such, represent important habitats for a wide variety of other organisms. As sessile organisms, brown algae require high levels of tolerance to various abiotic stressors such as osmotic pressure, temperature, and light. They differ from most terrestrial plants in many aspects of their biology, such as their ability to accumulate iodine [5], the fact that they are capable of synthesizing both C18 and C20 oxylipins [6], their use of laminarin as a storage polysaccharide [7], the original composition of their cell walls, and the associated cell wall synthesis pathways [8-10]. Many aspects of brown algal biology, however, remain poorly explored, presenting a high potential for new discoveries.

In order to fill this knowledge gap, *Ectocarpus siliculosus*, a small, cosmopolitan, filamentous brown alga (see [11] for a recent review) has been chosen as a model [12], mainly because it can complete its life cycle rapidly under laboratory conditions, is sexual and highly fertile, and possesses a relatively small genome (200 Mbp). Several genomic resources have been developed for this organism, such as the complete sequence of its genome and a large collection of expressed sequence tags (ESTs). Although *Ectocarpus *is used as a model for developmental studies [13,14], no molecular studies have been undertaken so far to study how this alga deals with the high levels of abiotic stress that are a part of its natural environment. This is also true for intertidal seaweeds in general, where very few studies have addressed this question.

In the 1960s and 1970s several studies (reviewed in [15]) examined the effects of abiotic stressors such as light, temperature, pH, osmolarity and mechanical stress on algal growth and photosynthesis. However, only a few of the mechanisms underlying the response to these stressors - for example, the role of mannitol as an osmolyte in brown algae [16,17] - have been investigated so far. Developing and applying molecular and biochemical tools will help us to further our knowledge about these mechanisms - an approach that was suggested 12 years ago by Davison and Pearson [18]. Nevertheless, it was only recently that the first transcriptomic approaches were undertaken to investigate stress tolerance in intertidal seaweeds. Using a cDNA microarray representing 1,295 genes, Collén *et al. *[19,20] obtained data demonstrating the up-regulation of stress-response genes in the red alga *Chondrus crispus *after treatment with methyl jasmonate [19] and suggesting that hypersaline and hyposaline stress are similar to important stressors in natural environments [20]. Furthermore, in the brown alga *Laminaria digitata*, Roeder *et al. *[21] performed a comparison of two EST libraries (sporophyte and protoplasts) and identified several genes that are potentially involved in the stress response, including the brown alga-specific vanadium-dependent bromoperoxidases and mannuronan-C5-epimerases, which are thought to play a role in cell wall modification and assembly. These studies have provided valuable information about the mechanisms and pathways involved in algal stress responses, but they were nevertheless limited by the availability of sequence information for the studied organisms at the time.

With the tools and sequences available for the emerging brown algal model *E. siliculosus*, we are now in a position to study the stress response of this alga on the level of the whole transcriptome. For this, we have developed an EST-based microarray along with several tools and annotations (available on our *Ectocarpus *transcriptomics homepage [22]), and used this array to study the transcriptomic response of *E. siliculosus *to three forms of abiotic stress: hyposaline, hypersaline, and oxidative stress. Hypersaline stress is a stress experienced by intertidal seaweeds - for example, in rockpools at low tide (due to evaporation) or due to anthropogenic influences - and is comparable to desiccation stress. Hyposaline stress is also common in the intertidal zone, and can arise, for example, due to rain. Furthermore, organisms with a high tolerance to saline stress can inhabit a wide range of habitats. *E. siliculosus *strains have been isolated from locations covering a wide range of salinity. A specimen was found in a highly salt-polluted area of the Werra river in Germany, where chloride concentrations at times reached 52.5 grams per liter [23]. At the same time, *E. siliculosus *can be found in estuaries, in the Baltic sea, and one strain of *E. siliculosus *was isolated from freshwater [24]. Oxidative stress is commonly experienced by living organisms. Reactive oxygen species (ROSs) are produced intracellularly in response to various stressors due to malfunctioning of cellular components, and have been implicated in many different signaling cascades in plants [25]. In algae, several studies have demonstrated the production of ROSs in response to biotic stress (reviewed in [26]). Therefore, protection against these molecules is at the basis of every stress response and has been well studied in many organisms. We simulated this stress by the addition of hydrogen peroxide to the culture medium.

## Results

### Determination of sub-lethal stress conditions

**Figure 1:**
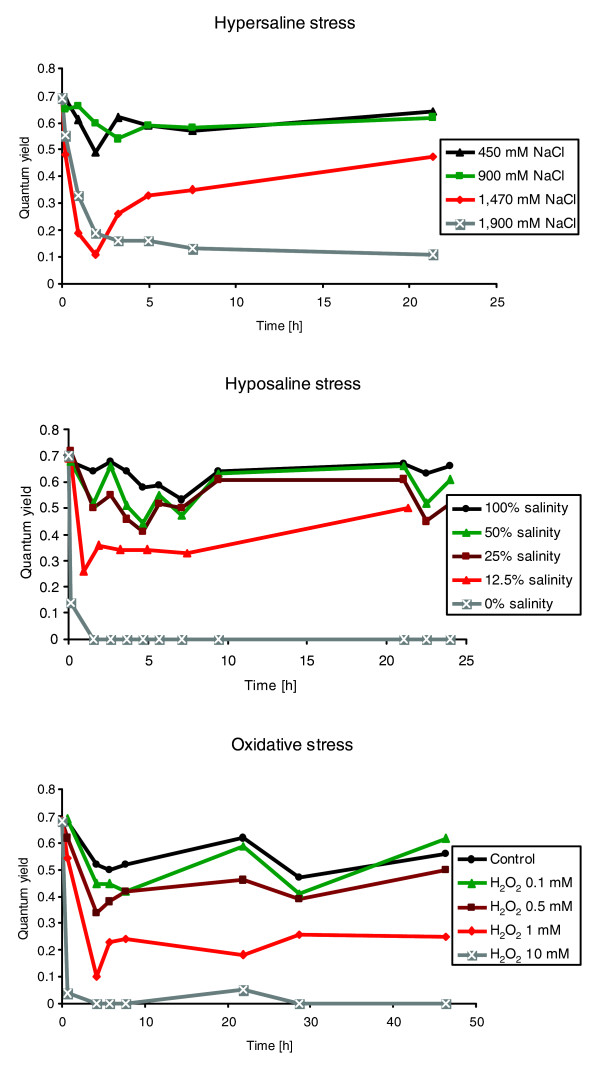
Effects of saline and oxidative stress of different intensities on the photosynthetic efficiency (quantum yield) of *E. siliculosus*. The conditions in red (1,470 mM NaCl, 12.5% seawater, and 1 mM H_2_O_2_) were the conditions chosen for the microarray analysis.

The aim of this study was to determine the mechanisms that allow short-term acclimation to abiotic stress. To be sure to monitor the short-term response to stress rather than just cell death, the intensity of the different stresses needed to be chosen carefully. Using a pulse amplitude modulation fluorometer (see Materials and methods), we measured the effects of different stress intensities on photosynthesis. Figure 1 shows the change in quantum yield of photosynthesis in response to different intensities of the different stresses, where values of over 0.5 indicate low stress. The quantum yield can vary during the course of the day even under controlled conditions, as changes in light have a strong impact on this parameter. Stress conditions were chosen to have a clear effect on the photosynthesis rate, but to be sub-lethal, allowing the alga to acclimate and recover. The conditions that corresponded best to these criteria were 1.47 M NaCl (hypersaline condition, approximately three times the salinity of normal seawater), 12.5% seawater, and 1 mM H_2_O_2 _(oxidative stress condition), although, for this last stressor, we can assume that the H_2_O_2 _concentration in the medium decreases over the course of the experiment. Each stress was applied for 6 hours because this corresponds to the time span between high and low tide. In addition, experiments carried out on land plants [27] and red algae [19] have indicated that the application of stress for 6 hours induces the most marked changes in transcription.

Initially, we had considered a fourth stress condition, 2 M sorbitol in artificial sea water (ASW), to imitate the osmotic pressure of the hypersaline treatment without the possible effects of the salts. However, this treatment was not included in the final experiment because cultures did not survive this treatment for 6 hours. For the other stresses, we observed 100% recovery of photosynthesis after about 6 days, even after 24 hours of stress (Additional data file 1).

### Intracellular osmolarity and Na^+ ^concentration

**Figure 2:**
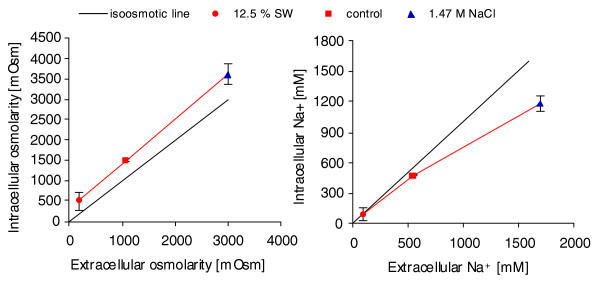
Intracellular versus extracellular osmolarity and Na^+ ^concentration under saline stress. Oxidative stress samples are not shown as they did not differ significantly from the control sample. Every point represents the mean of five biological replicates ± standard deviation.

Apart from the photosynthetic activity, we also measured intracellular osmolarity and Na^+ ^concentrations (Figure 2). After 6 hours of exposure to different salinities, the intracellular osmolarity was always about 500 mOsm higher than that of the extracellular medium. The intracellular Na^+ ^concentration was about 500 mM lower than in the extracellular medium under hypersaline stress, 60 mM lower under control conditions, and the same under hyposaline stress. Oxidative stress had no detectable effect on the intracellular ion composition or osmolarity (data not shown).

### The *E. siliculosus *microarray represents 17,119 sequences

We designed a microarray based on 90,637 ESTs obtained by sequencing clones from 6 different cDNA libraries: immature sporophyte (normalized and non-normalized), mature sporophyte, immature gametophyte, mature gametophyte, and stress (sporophyte). Cleaning and assembly resulted in the generation of 8,165 contigs and 8,874 singletons. In addition, 21 genomic sequences and 231 *E. siliculosus *Virus 1 (EsV-1) genes were included. The array design file has been deposited under the accession number [ArrayExpress:A-MEXP-1445] and is also available on our *Ectocarpus *transcriptomics homepage [22].

Of the 17,119 genes represented on the array, 12,250 gave a significant signal over background in our experiments and were considered to be expressed under the conditions tested. The analysis focused on these 12,250 genes (see Materials and methods). A first comparison with the data obtained from a tiling experiment with *E. siliculosus *(MP Samanta and JM Cock, personal communication), where 12,600 genes were considered strongly expressed, demonstrates that our array offers a rather complete coverage of at least the highly transcribed parts of the *E. siliculosus *genome, suggesting that we are working at the whole genome scale.

### cDNA synthesis and amplification provided consistent results with both mRNA and total RNA samples

For reasons as yet unknown, cDNA synthesis reactions with *E. siliculosus *are inhibited at high concentrations of RNA. Therefore, we decided to synthesize cDNAs from a small quantity of total RNA or mRNA, and to include a PCR amplification step in the protocol to obtain sufficient double-stranded cDNA (4 μg) for each hybridization. A comparison of the four four-fold replicates synthesized from 30 ng of mRNA and the single four-fold replicate synthesized from 100 ng total RNA showed that these two protocols yielded similar results. All total RNA replicates clustered with the mRNA replicates of the same stress (data not shown). Nevertheless, at a false discovery rate (FDR) of 5%, 163 transcripts gave significantly different results with the two types of sample. These transcripts represented mainly constituents of the ribosome, as revealed by a Kyoto Encyclopedia of Genes and Genomes (KEGG) Orthology Based Annotation System (KOBAS) analysis and by an analysis of overrepresented GO terms (Additional data file 2).

### Validation of microarray results using quantitative PCR

Nineteen genes that exhibited significant changes in their expression patterns in the microarray analysis were analyzed by real time quantitative PCR (RT-qPCR). Eighteen of these had similar expression profiles in both the microarray experiment and the RT-qPCR experiment (correlation coefficient r of between 0.57 and 0.99; Table 1). Only one gene, which codes for a microsomal glutathione S-transferase, displayed a different pattern in the two experiments (r = -0.48). Furthermore, the seven most stable 'housekeeping genes' as identified by qPCR in a previous report [28] showed only statistically non-significant relative changes of <1.5-fold (log2-ratio <0.58) in the microarray experiment (Table 2). This demonstrated that the protocol for cDNA amplification provided reliable measures of the relative transcript abundances. Although this method has been successfully applied in several small-scale expression studies [29-35], to our knowledge, the use of this technique has not been reported with commercial photolithographically synthesized arrays.

**Table 1:** Comparison of microarray and RT-qPCR results for genes changing expression

ID	Genome ID	Name	r	Function
CL4038Contig1	[Esi0355_0025]	*HSP70*	0.87	HSP70
LQ0AAB7YD09FM1.SCF	[Esi0155_0065]	*NADH*	0.94	NADH dehydrogenase
CL7513Contig1	[Esi0269_0011]	*ProDH*	0.79	Proline dehydrogenase
CL3741Contig1	[Esi0024_0066]	*TF*	0.90	Putative transcription factor
LQ0AAB12YN05FM1.SCF	[Esi0399_0008]	*WD_rep*	0.66	WD repeat gene
CL1Contig3	[Esi0085_0055]	*CLB1*	0.95	Chlorophyll binding protein
CL43Contig1	[Esi0199_0054]	*CLB2*	0.98	Fucoxanthin binding protein
CL7742Contig1	[Esi0026_0055]	*TagS*	0.69	TAG synthase
CL2765Contig1	[Esi0526_0006]	*NH4-Tr*	0.96	Ammonium transporter
CL3832Contig1	[Esi0437_0012]	*FOR*	0.67	Phycoerythrobilin:ferredoxin oxidoreductase
LQ0AAA16YN10FM1.SCF	[Esi0153_0004]	*Arg-MetTr*	0.71	Arginine N-methyltransferase
CL7099Contig1	[Esi0018_0111]	*HSD*	0.83	Homoserine dehydrogenase
CL6576Contig1	[Esi0107_0059]	*IGPS*	0.97	Indole-3-glycerol-phosphate synthase
CL7231Contig1	[Esi0686_0001]	*CDPK*	0.85	cAMP-dependent protein kinase
CL4027Contig1	[Esi0122_0054]	*mGST*	-0.48	Microsomal glutathione S-transferase
CL4274Contig1	[Esi0023_0183]	*SNR*	0.57	SNR (vesicular transport)
CL5850Contig1	[Esi0109_0088]	*mG*	0.99	Glycin-rich protein
CL455Contig1	[Esi0159_0021]	*G6PD*	0.91	Glucose-6-phosphate 1-dehydrogenase
CL6746Contig1	[Esi0116_0065]	*IF4E*	0.91	Eukaryotic initiation factor 4E

R is the Pearson correlation coefficient between the microarray and the RT-qPCR expression profile. ID corresponds to the name of the sequence on the array.

**Table 2:** Comparison of microarray and RT-qPCR results for housekeeping or stable genes

ID	Genome ID	Name	Maximum change ARRAY	Maximum change QPCR	Function
LQ0AAB30YA12FM1.SCF	[Esi0298_0008]	Dyn	0.23	0.77	Dynein
CL1914Contig1	[Esi0021_0024]	ARP2.1	0.22	0.44	Actin related protein
CL3Contig2	[Esi0387_0021]	EF1A	0.08	0.46	Elongation factor 1 alpha
CL8Contig12	[Esi0053_0059]	TUA	0.57	0.91	Alpha tubulin
CL1073Contig1	[Esi0054_0059]	UBCE	0.22	0.38	Ubiquitin-conjugating enzyme
CL29Contig4	[Esi0302_0019]	UBQ	0.18	0.82	Ubiquitin
CL461Contig1	[Esi0072_0068]	R26S	0.22	n/a	Ribosomal protein S26

The table displays the maximum log2-ratio between any stress and the control condition for both the microarray and the RT-qPCR analysis. No RT-qPCR value is available for R26S, as this gene was used for normalization of the RT-qPCR samples. ID corresponds to the name of the sequence on the array.

### Ribosomal protein genes are among those whose transcript abundances are least affected by stress

The 100 most stably expressed genes in these microarray experiments included 51 genes with unknown functions. Nineteen genes code for ribosomal proteins, and 21 genes are known housekeeping genes with functions related to protein turnover (transcription, 4 genes; translation, 3 genes; degradation, 3 genes), energy production (6 genes), and the cytoskeleton (5 genes). For a detailed list of these most stably expressed genes, please see Additional data file 3.

### Classification of stress response genes using automatic annotations

**Figure 3:**
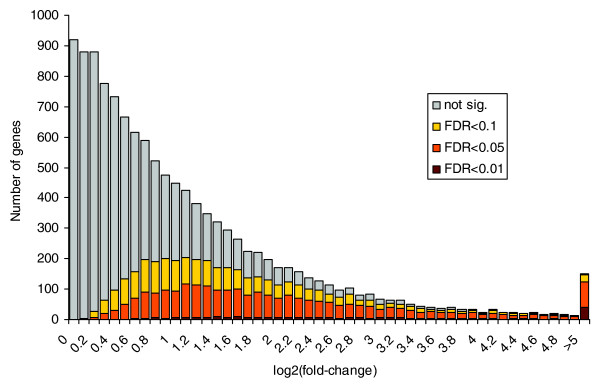
Distribution of observed fold-changes (log2-ratios of stress and control samples). All three comparisons between stress and control treatments were considered and the observed frequencies averaged. The color coding shows how many transcripts were labeled as differentially expressed at different FDRs. Not sig., not significant.

Overall, 8,474 genes were identified as being differentially expressed in at least one of the conditions compared to the control, allowing a FDR of 10% (5,812 were labeled significant at an FDR of 5%). As can be seen in Figure 3, the relative change for these genes ranged from 1.2-fold (log2-ratio ≈0.3) to more than 32-fold (log2-ratio >5). Of these 8,474 genes, 2,569 (30%) could be automatically annotated with GO terms using the GO-term Prediction and Evaluation Tool (GOPET) [36] and 1,602 (19%) with KEGG orthology annotations using the KOBAS software [37]. These automatic annotations were analyzed for each stress condition individually, to identify GO categories and KEGG pathways that were significantly over-represented.

**Figure 4:**
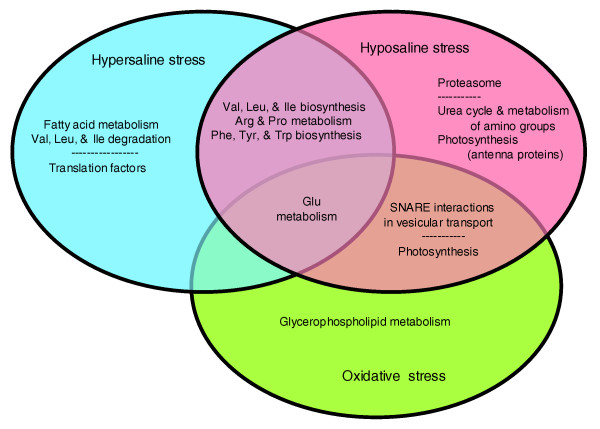
Venn diagram of KEGG pathways identified as over-represented among the transcripts significantly up- or down-regulated (FDR <0.1) in the different stress conditions. Only KEGG pathways with q-values < 0.1 in at least two conditions or for both datasets (FDR of 0.05 and FDR of 0.1) were considered. The general category 'other enzymes' was not included. Further 'SNARE interactions in vesicular transport' includes the category 'SNARE', and 'photosynthesis' includes 'photosynthesis proteins' and 'porphyrin and chlorophyll metabolism'. No pathways were found to be common only to hyposaline and hypersaline stress. SNARE, soluble N-ethylmaleimide-sensitive factor attachment receptor.

The KOBAS results (Figure 4; Additional data file 4) indicated that under hyposaline and hypersaline stresses most of the changes involved down-regulation of the synthesis and metabolism of amino acids. More precisely, genes involved in the synthesis of valine, leucine, and isoleucine, as well as that of the aromatic amino acids (phenylalanine, tyrosine, tryptophan), and arginine and proline metabolism were affected. This effect on amino acid synthesis was less marked for oxidative stress, where glutamate metabolism was the only amino acid metabolism affected. Under hypersaline conditions, there was also an increase in transcripts coding for enzymes that metabolize valine, leucine, and isoleucine. In addition, photosynthesis and vesicular transport seemed to be altered by both hyposaline and oxidative stress. Pathways that appeared to be specifically affected by one stress included the up-regulation of fatty acid metabolism and down-regulation of translation factors under hypersaline stress, the up-regulation of the proteasome and down-regulation of nitrogen metabolism under hyposaline stress, and an increase in glycerophospholipid metabolism under oxidative stress (Figure 4). A complete list of the pathways identified is available in Additional data file 4, with possible artifacts arising from the automatic annotation marked in grey.

The GOPET analysis (Table 3; Additional data file 5) was focused on the molecular function of the individual genes rather than their role in a specific pathway. Only three GO terms were identified as being over-represented among the up-regulated genes: arginase and agmatinase activity under hypersaline conditions, and microtubule motor activity under oxidative stress. Most GO terms were found to be significantly over-represented among the down-regulated genes. In agreement with the down-regulation of amino acid metabolism identified by the KOBAS analysis, we observed a decrease in the abundance of transcripts encoding aminoacyl-tRNA ligases in hypersaline and hyposaline conditions using the GOPET annotations. Also, under hypersaline stress, we observed down-regulation of genes associated with the GO terms RNA binding and translation factor activity, which corresponds to the KEGG category translation factors, and down-regulation of transcripts coding for proteins with a CTP synthase activity, which are involved in purine and pyrimidine metabolism. Under hyposaline stress, we observed that NAD(P)^+ ^transhydrogenases, a number of transferases and oxidoreductases involved in amino acid metabolism, as well as genes with functions in nucleic acid and chlorophyll binding, were most affected, the latter matching well with the pathways 'photosynthesis-antenna proteins' identified by KOBAS. Under oxidative stress, using the GOPET annotations, we detected down-regulation of several different categories of transferases, nitrate transporters, oxidoreductases involved in steroid metabolism, and 3-isopropylmalate dehydratase-like enzymes that are involved in amino acid metabolism. Here, the KOBAS analysis did not identify any significantly up- or down-regulated pathways. Also in contrast to the KOBAS results, no GO terms were significantly over-represented among the genes identified as being up- or down-regulated in both oxidative and hypersaline stresses, or in all three stresses at the same time.

**Table 3:** GO terms identified to be over-represented among the transcripts of significantly up- or down-regulated in the different stress conditions

Condition	Change in expression	Category	Function	GO ID
Hyper	Down	Nucleic acid binding	RNA binding (mRNA, rRNA, snoRNA)	[GO:0003723]; [GO:0003729]; [GO:0019843]; [GO:0030515]
		Nucleic acid binding	Translation factor activity (elongation and initiation)	[GO:0008135]; [GO:0003746]; [GO:0003743]
		Lyase	UDP-glucuronate decarboxylase activity	[GO:0048040]
		Ligase	CTP synthase activity	[GO:0003883]
		Isomerase activity	Intramolecular oxidoreductase activity	[GO:0016860]
	Up	Hydrolase	Agmatinase activity	[GO:0008783]
		Hydrolase	Arginase activity	[GO:0004053]
Hyper Hypo	Down	Ligase	Aminoacyl-tRNA ligase activity (inlcuding Pro, Ser, Ile, Glu)	[GO:0004812]; [GO:0016876]; [GO:0004828]; [GO:0004829]; [GO:0004822]
				
Hypo	Down	Oxidoreductase (S, peroxide)	Antioxidant activity (glutathione-disulfide reductase and catalase, cytochrome-c peroxidase)	[GO:0016209]; [GO:0004362]; [GO:0004096]; [GO:0004130]
		Nucleic acid binding	Structure-specific DNA binding	[GO:0000404]; [GO:0032134]; [GO:0000403]; [GO:0032137]; [GO:0032138]; [GO:0032139]
		Tetrapyrrole binding	Chlorophyll binding	[GO:0016168]
		Lyase	Carbon-oxygen lyase activity	[GO:0016835]
		Transferase (N)	Transaminase activity (including TYR, ASP, histidinol-P, aromatic amino acids)	[GO:0008483]; [GO:0004838]; [GO:0004400]; [GO:0008793]; [GO:0004069]
		Transferase (C1)	Aspartate carbamoyltransferase activity	[GO:0004070]
		Transferase (glycosyl)	Transferase activity, transferring pentosyl groups	[GO:0016763]
		Oxidoreductase CH-CH	Biliverdin reductase activity	[GO:0004074]
		Oxidoreductase (CH-NH2)	Glutamate synthase activity	[GO:0015930]
		Isomerase	Isomerase activity	[GO:0016853]
		Transporter	NAD(P)+ transhydrogenase (B-specific) activity	[GO:0003957]
				
Hypo	Down	Oxidoreductase	Oxidoreductase activity	[GO:0016491]
Oxi			Oxidoreductase activity, acting on NADH or NADPH	[GO:0016651]; [GO:0016652]
			Oxidoreductase activity, acting on the CH-OH group of donors, NAD or NADP as acceptor (including L-iditol 2-dehydrogenase activity)	[GO:0016616]; [GO:0016614]; [GO:0003939]
Oxi	Down	Lyase	3-Isopropylmalate dehydratase activity	[GO:0003861]
		Transferase (P)	Amino acid kinase activity	[GO:0019202]
		Transporter	Nitrate transmembrane transporter activity	[GO:0015112]
		Transferase (C1)	S-adenosylmethionine-dependent methyltransferase activity (including nicotinate phosphoribosyltransferase)	[GO:0008757]
		Oxidoreductase (steroids)	Steroid dehydrogenase activity, acting on the CH-OH group of donors, NAD or NADP as acceptor	[GO:0033764]
		Transferase (glycosyl)	Transferase activity, transferring pentosyl groups	[GO:0016763]; [GO:0004853]
		Transferase (glycosyl)	Uracil phosphoribosyltransferase activity	[GO:0004845]
		Transferase (P)	Phosphoribulokinase activity	[GO:0008974]
	Up	Motor activity	Microtubule motor activity	[GO:0003777]

The table shows only pathways that were labeled significant at an FDR <10% in both sets of significant genes (5% FDR and 10% FDR).

### Manual classification of stress response genes with the most significant changes in expression

To identify the most important mechanisms involved in the stress response, we manually classified and examined in detail 966 genes that exhibited the most significant changes in one of the stress conditions compared to the control (that is, genes that meet both criteria: significance at an FDR <1% and a relative change in expression of more than two-fold). A complete list of these genes, including their putative function, assigned manually based on sequence homology of the corresponding genome sequence to public protein databases, can be found in Additional data file 3.

We identified 519 genes (53.7%) with no homologues in either the National Center for Biotechnology Information (NCBI) databases or other heterokont genomes (e-value > 1e-10). An additional 122 genes (12.6%) code for conserved genes with unknown function. Of these 122 conserved genes, 23 (18.9%) are conserved only within the heterokont lineage. The remaining 325 genes (33.6%) were divided into 12 groups according to their putative functions in amino acid metabolism, DNA replication and protein synthesis, protein turnover, carbohydrate metabolism, photosynthesis-related processes, fatty acid metabolism, transporters, vesicular trafficking and cytoskeleton, classical stress response pathways, autophagy, signaling, and other processes. The following section gives a brief overview of the different groups of genes identified among the most significantly regulated genes.

Among genes involved in amino acid metabolism, we found a total of 32 down-regulated genes related to the metabolism of all 20 standard amino acids except aspartic acid. In contrast, nine genes were induced in at least one abiotic stress condition. These were involved in the metabolism of proline, arginine, cysteine, alanine, phenylalanine, tyrosine, tryptophan, leucine, isoleucine, and valine. Highly regulated genes involved in the different steps of DNA replication and protein synthesis coded for proteins, including helicases, DNA polymerases and related enzymes, proteins involved in purine and pyrimidine synthesis, DNA repair proteins, transcription factors, RNA processing enzymes, proteins involved in translation, ribosomal proteins, and proteins for tRNA synthesis and ligation. Most of these genes were down-regulated in all stress conditions, but some genes were up-regulated in response to abiotic stress. These genes include some helicases, transcription factors, and DNA repair proteins. We also found seven genes related to protein turnover to be down-regulated and six to be up-regulated in one or more of the stress conditions. Among the up-regulated genes, there were two ubiquitin conjugating enzymes, which play a potential role in targeting damaged proteins to the proteasome, or control the stability, function, or subcellular localization of proteins.

The situation was similar for genes involved in carbohydrate metabolism, where we found both glycolysis- and citric acid cycle-related genes to be strongly down-regulated under all the stresses tested (six and seven genes down-regulated, respectively). However, four genes, encoding a gluconolactonase, a xylulokinase, a phosphoglycerate kinase, and an isocitrate lyase, were up-regulated. In particular, an isocitrate lyase gene was 19- to 212-fold up-regulated under the different stress conditions. Photosynthesis-related genes that were regulated in response to abiotic stress included eight chlorophyll a/c binding proteins as well as genes responsible for the assembly of photosystem 2, electron transport, light sensing, and carotenoid synthesis. Many of these genes were strongly affected in the hypersaline condition, with the majority being down-regulated (17 versus 11 that were up-regulated). There was at least one gene that was up-regulated under one or more stress condition in every group. Genes with roles in fatty acid metabolism altered their expression patterns in a similar way under all stress conditions. We were able to distinguish between two groups: three genes involved in the synthesis of fatty acids, which were down-regulated; and genes functioning in the degradation of fatty acids, among which five of six genes were up-regulated. We further observed that three genes involved in lipid synthesis were up-regulated, and genes involved in inositol metabolism were also affected.

With respect to transporters, we identified five genes encoding nitrogen transporters (all down-regulated) as well as three genes encoding sugar transporters (all up-regulated). Genes coding for ion transporters were also mainly down-regulated under hypersaline and hyposaline conditions, although two potassium and magnesium transporter genes were up-regulated under hypersaline stress. Among genes responsible for the transport of solutes and proteins to the mitochondrion, we observed an up-regulation mainly in the hyposaline stress condition. Regarding genes related to vesicular trafficking and the cytoskeleton, we identified 13 up- and 6 down-regulated genes, many of these genes containing an ankyrin repeat domain and showing strongest changes in transcription under hyposaline and oxidative stress conditions.

We further found several classical stress response genes to be up-regulated. Four genes coding for heat shock proteins (HSPs) were up-regulated mainly under hyposaline and oxidative stress, but there were also two genes coding for a chaperonin cpn60 and a prefoldin, each of which was down-regulated. In addition, we found genes involved in protection against oxidative stress to be induced. These include a glutaredoxin (oxidative stress), a methionine sulfoxide reductase (hyposaline stress), and three glutathione peroxidases (mainly hypersaline stress). At the same time, however, a catalase-coding gene was down-regulated in all stress conditions, most strongly under hyposaline stress.

Two genes involved in autophagy, one of which is represented by two sequences on the microarray, were up-regulated in all stress conditions and several genes with putative signaling functions were affected. Six protein kinases were among the most significantly up-regulated genes: three equally under all stress conditions, and one each specifically under hyposaline, hypersaline and oxidative stress. Furthermore, one protein kinase and one WD-40 domain containing gene were down-regulated under hyper- and hyposaline stress, respectively.

Several other genes are not mentioned here, either because only a very vague prediction of their function was possible, or because they are difficult to put into categories with other genes. More detailed information about these genes can be found in Additional data file 3.

### Stress response genes with unknown functions

All unknown and conserved unknown genes present among the most significantly regulated genes were sorted into groups according to their sequence similarity (Additional data file 6). Among the groups with three or more members, there were three (I to III) that had no known homologs in species other than *E. siliculosus*, and three (IV to VI) for which we were able to find homologs in other lineages for most of the sequences. A more detailed description of all of the unknown and unknown conserved stress response genes, including an analysis of conserved protein and transmembrane domains, is available in Additional data file 6.

### Known brown algal stress genes

Many of the brown alga-specific stress response genes identified in *L. digitata *by Roeder *et al. *[21] were not among the most regulated genes identified in this study. Nevertheless, we decided to examine their expression patterns in more detail. The array used in this study contained probes for one vanadium-dependent bromoperoxidase (CL83Contig2), but this gene was not strongly regulated under the different stress conditions (1.06-fold to 1.4-fold induced, *P *= 0.75). Twenty-four C5-epimerases were represented, but none of these genes were among the most significantly regulated loci, although several of them were either induced or repressed under the different stress conditions. A detailed list of these genes, including their expression profiles, can be found in Additional data file 7. Finally, we decided to consider genes involved in the synthesis of mannitol, a well-known osmolyte in brown algae [16,17]. Only one enzyme specific to the synthesis of this polyol could be clearly identified based on sequence homology: mannitol 1-phosphate dehydrogenase (see [38] for a description of the mannitol synthesis pathway in brown algae). Our array contains probes for two genes identified as potential mannitol 1-phosphate dehydrogenases: one (CL200Contig2 corresponding to Esi0017_0062 in the *Ectocarpus *genome), which was among the most significantly regulated genes and six-fold down-regulated in hyposaline condition, and one (CL2843Contig corresponding to Esi0020_0181), which was generally expressed at a very low level but was up-regulated approximately five-fold under hypersaline stress (*P *= 0.066).

### Clusters of genes with similar expression patterns

**Figure 5:**
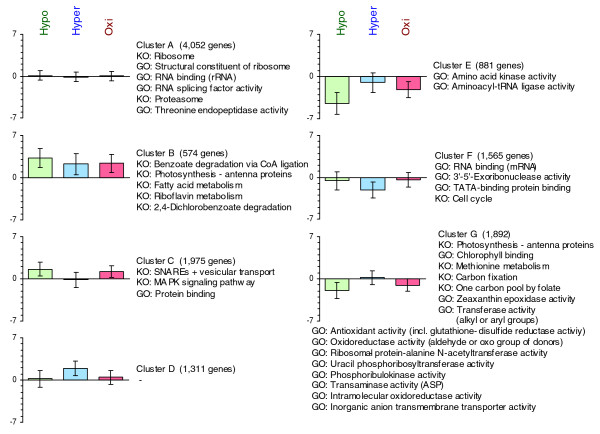
Expression graphs of clusters identified by the k-means algorithm. The graphs display the log2-ratio of all stress conditions (hypo = green, hyper = red, oxi = blue) with the control condition. GO terms and KEGG pathways (KO) identified as over-represented in these clusters (FDR = 10%) are shown next to the graph.

Based on a figure of merit (FOM) graph, we decided to divide the set of expressed genes into seven different clusters (A to G). These clusters, along with the GO terms and KEGG pathways that are over-represented among each of them, are shown in Figure 5. We identified one cluster (A) representing the stably expressed genes, three clusters included mainly up-regulated genes (B-D), and the remaining three clusters included mainly down-regulated genes (E-G). Among both the up- and down-regulated clusters, we found one cluster each that was equally affected by all stress conditions (B and E), one each where gene expression was affected only by hyposaline and oxidative stress conditions (C and G), and one cluster each where gene expression was affected mainly by hypersaline stress (D and F). Most of the principal functions identified for each cluster by GOPET and KOBAS fit well with the results from our earlier analysis of the up- and down-regulated genes.

## Discussion

This study presents the first global gene expression analysis of a brown alga. Our goal was to determine the transcriptomic changes in response to short-term hypersaline, hyposaline and oxidative stress - three stresses that play an important role in the natural habitat of many brown algae, the intertidal zone [20,26]. Our results show that almost 70% of the expressed genes had a modified expression pattern in at least one of the examined stress conditions. This is in contrast to what has been observed in flowering plants, where the proportion of significantly regulated genes generally ranges from 1% to 30%, depending on types of abiotic stress examined, their number, and the statistical treatment applied (see [27,39,40] for some examples). Our findings demonstrate that, rather than relying on a few specific stress response proteins, *E. siliculosus *responds to abiotic stress by extensive reprogramming of its transcriptome.

**Figure 6:**
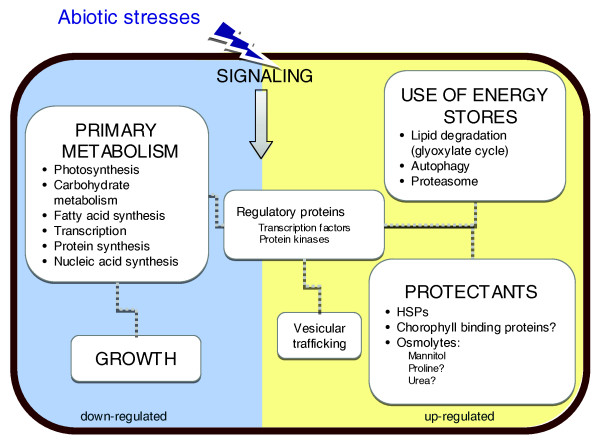
Major transcriptomic changes in *E. siliculosus *under short-term oxidative and saline stress. This schema summarizes the most important transcriptomic changes discussed in the text. Processes on the left (blue) were repressed, while processes on the right (yellow) were activated. Please note that this graph displays only the general trends; some of the pathways are not regulated in all stress conditions and not all genes of one pathway are always regulated in the same way (see text for details).

A more detailed analysis of the manual annotation of the 966 most significantly regulated genes and the results for the GOPET and KOBAS analysis for all three stress conditions, reveals two major themes concerning the short-term stress response of *E. siliculosus*: down-regulation of primary metabolism and growth processes; and activation of energy stores and of genes and pathways involved in 'stress management'. These findings are summarized in Figure 6. In the following sections we will first discuss the differences observed between the different stress conditions that were tested, and then highlight some of the general trends that emerged from our data.

### Comparison of stress conditions

We have compared each stress condition to the control condition, and analyzed these results using KOBAS and GOPET. Due to the necessity to control the FDR with multiple testing, the chance of beta-errors (that is, the chance of falsely labeling a gene or a pathway as not significantly regulated) greatly increased, making a direct comparison of the genes and groups of genes identified as being up- or down-regulated under the different stress conditions prone to false conclusions. Therefore, we based our comparison of the different stresses on the cluster analysis and the results from the manually analyzed 966 most significantly changing genes.

The first and most apparent observation from the clustering was that the changes in gene expression induced by hyposaline and oxidative stress were more similar to each other than to those observed under hypersaline stress. One explanation for this might be that hypersaline stress, although it is a common stressor in the natural habitat of brown algae, is not likely to occur at the same intensity in the field as that used for our laboratory experiments (about three times the concentration of normal seawater). Even though we did not observe a strong difference in the efficiency of photosynthesis under the different stress conditions, it is possible that hypersaline stress, at the intensities applied in our experiments, represents a condition the alga is less able to adjust to. This hypothesis is supported by the fact that in cluster F (down-regulated in hypersaline conditions) cell cycle-related genes were over-represented, indicating that growth was most strongly affected under hypersaline stress conditions. Furthermore, other classical stress responses, such as the up-regulation of SNARE (soluble N-ethylmaleimide-sensitive factor attachment receptor)-related genes, which are important for cellular transport of vesicles and their fusion with membranes and play a role in plant development and abiotic stress response [41], were observed mainly under oxidative and hyposaline stress.

There are also several smaller differences between oxidative and hyposaline stress, such as the induction of a glutaredoxin gene (Additional data file 2) under oxidative stress conditions. Glutaredoxins are known to play a central role in the protection against oxidative damage [42], as they can be oxidized by diverse substrates, including ROSs, and are reduced by glutathione. Other genes did not change expression under oxidative stress, but were specifically regulated under saline stress. Such examples are given in the following paragraphs.

### Down-regulation of growth and primary metabolism

Many genes involved in several pathways related to growth and primary metabolism were identified to be down-regulated by more than one of our analyses (GOPET, KOBAS, k-means clustering, and manual analysis). We observed a decrease in the abundance of transcripts of genes that are important in the synthesis of purine and pyrimidine nucleotides and, correspondingly, of several genes responsible for the replication of DNA. This function is essential for cell division, a process affected in all of the stress conditions examined.

A reduction in growth implies a reduction in the requirements for primary metabolites necessary to fuel this growth. Within our dataset, we found widespread evidence for down-regulation of processes involved in primary metabolism. This was most pronounced in the case of protein synthesis. Several genes encoding enzymes involved in this and related processes (the synthesis of amino acids, their ligation with the appropriate tRNAs, the production of mRNA (that is, transcription), and the actual assembly of polypeptide chains (that is, translation)) were down-regulated under stress conditions. Furthermore, genes responsible for the uptake of nitrogen, which is used mainly for the synthesis of amino acids, were also down-regulated. Together, this provides a strong indication that the overall rate of protein synthesis was reduced under the stress conditions examined.

However, there were additional primary metabolic processes that were at least partially affected by the stress treatments. These included the synthesis of fatty acids, photosynthesis and pigment synthesis, and carbohydrate metabolism. Again, these changes probably reflected a decreased need for metabolites for growth.

One possible explanation for the observed down-regulation of genes involved in growth and primary metabolism can be found in the results of our pulse amplitude modulation fluorometer experiment: we observed that the efficiency of photosynthesis decreased almost immediately after the application of the stress treatments. Photosynthesis is strongly affected by environmental stress [43]. A decrease in photosynthetic efficiency is synonymous with a decrease in energy production, and we can assume that reducing all of these aspects of primary metabolism might represent a means of conserving energy. This phenomenon is known in higher plants, where Kovtun *et al. *[44] have reported cross-talk between oxidative stress and auxin signaling, auxin being a major growth hormone in higher plants. Most likely, this cross-talk allows plants to shift their energy from growth-related processes to stress protection and survival. This might also be true for *E. siliculosus*, where the observed down-regulation of growth- and primary metabolism-related genes might represent a way of compensating for reduced energy production under stress conditions and redirecting energy to specific stress response processes.

### Activation of protein degradation, energy stores, and nutrient recycling

We observed an up-regulation of two genes involved in autophagy in all three of the stresses examined. Under nutrient-limited and under stress conditions, this process has been shown to play a role in the re-allocation of sugar and nutrients to essential biological processes in several organisms [45]. The activation of autophagy-related genes might, therefore, just like the down-regulation of growth-related processes, represent a mechanism of compensating for reduced energy production under stress conditions and provide sugars and nutrients for both core biological processes and synthesis of stress proteins. This coincides with the strong up-regulation of an isocitrate lyase gene under all stress conditions. Isocitrate lyases are enzymes located in the glyoxysome and catalyze a rate-controlling step in the glyoxylate cycle (reviewed in [46]), one function of which is the conversion of lipids to carbohydrates when non-lipid-derived storage reserves are depleted. Corresponding to this and the up-regulation of autophagy-related genes, we observed an up-regulation of three genes coding for sugar transporters in all stress conditions and of genes coding for mitochondrial exchange proteins under the hyposaline and oxidative stress conditions. These transporters may direct recycled sugars and nutrients to the mitochondrion, where they can be used for energy production.

Recently, additional roles of autophagy have been discovered in higher plants, including the degradation and removal of oxidized or damaged proteins during stress [47]. To a certain degree, these roles overlap with the role of the proteasome. Although we found some genes involved in protein turnover to be down-regulated, the KEGG pathway 'proteasome' was identified as up-regulated in the hyposaline stress condition and we identified two genes involved in ubiquitination among the most significantly up-regulated genes under all stress conditions. Ubiquitination is a process in which proteins are labeled with a small polypeptide (ubiquitin) [48], thereby modifying their stability, function, or subcellular localization. This could serve regulatory purposes (for example, by targeting transcription factors or other regulatory proteins for degradation by the proteasome), accelerate translation of the large-scale transcriptomic changes into changes in protein abundance, and/or, just like autophagy, facilitate nitrogen recycling [49].

### Activation of signaling pathways

Large-scale transcriptomic reprogramming as observed in our dataset most certainly requires a large number of signals for coordination. We have already discussed a possible regulatory role of ubiquitination in the previous paragraph. This, however, is not the only regulatory mechanism highlighted by the transcriptomic changes in our dataset. We found several other genes that might play roles in orchestrating the abiotic stress response of *E. siliculosus*. For example, three protein kinases with a potential role in cell signaling were strongly up-regulated under all stress conditions, while three other members of this large family appeared each to be specific to one particular stress. Furthermore, several potential transcription factors were strongly regulated under different stress conditions. Since there is still very little known about the molecules and proteins involved in the stress sensing signaling cascades of brown algae, these genes provide particularly interesting candidates for more targeted experiments such as targeted mutagenesis and chromatin immunoprecipitation in the case of the putative transcription factors.

In addition to transcription factors and protein kinases, we detected an up-regulation of genes involved in fatty acid metabolism, and more specifically fatty acid catabolism. Fatty acid derivates such as oxylipins have been shown to function in signaling in both terrestrial plants [50] and marine algae [51]. Thus, we can conclude that our data show an up-regulation of genes putatively involved in several different signaling pathways.

### Synthesis of 'classical' stress response proteins

The only medium throughput transcriptomic analysis of the abiotic stress response in brown algae so far [21], conducted with protoplasts of *L. digitata*, reported the transcriptional activation of vanadium-dependent bromoperoxidases and C5-epimerases. Neither of these genes was regulated in our study. While in *L. digitata *vanadium-dependent bromoperoxidases (enzymes implicated in the synthesis of halogenated organic compounds associated with defense of seaweeds against biotic stressors [52]) comprise a multigenic family [21], the *E. siliculosus *genome contains only a single copy of a vanadium-dependent bromoperoxidase, indicating a possibly different or subordinate role in this organism. Regarding C5-epimerases, which are enzymes responsible for the modification of brown algal cell walls [10] highly represented in the *Ectocarpus *genome, we observed differences in regulation between the study of Roeder *et al. *[21] and our study. This can be explained by the nature of the stress (generation of protoplasts (that is, removal of the cell wall) in [21] versus milder saline or oxidative stress in our study).

Generally, only a few 'classical' stress response genes changed expression in our experiments. In most organisms, up-regulation of genes coding for HSPs and other chaperones has been observed under abiotic stress conditions. These molecules stabilize proteins and membranes, and have been shown to play a vital role in protecting against stress by re-establishing normal protein conformation and, thus, cellular homeostasis [53]. In *E. siliculosus*, we observed four HSPs or chaperones to be among the most significantly up-regulated genes in hyposaline and oxidative stress conditions. However, this was not the case under hypersaline stress conditions. Moreover, two chaperone-like proteins were down-regulated under all stress conditions. The situation was similar for genes coding for proteins with antioxidant activity. Three genes coding for glutathione peroxidases were up-regulated under hypersaline and oxidative stress, and one glutaredoxin under hyposaline stress. At the same time, two genes encoding a glutathione S-transferase and a catalase were among the most significantly down-regulated in all stress conditions. Consequently, the GO term 'antioxidant activity' was significantly over-represented among the most down-regulated genes in the hyposaline condition. In flowering plants all of these proteins are known to carry out important functions in the protection against reactive oxygen species [25]. Our finding that these genes were not induced was, at first, surprising, but is in accordance with a transcriptomic analysis of the abiotic stress response of the intertidal red alga *C. crispus*. Collén *et al. *[20] have reported that the average expression of HSP-coding genes was only moderately elevated (approximately 1.3-fold) under hypersaline and hyposaline conditions. Furthermore, in *E. siliculosus*, the average expression of genes coding for proteins with antioxidant activity was slightly repressed (1.17-fold) under hyposaline stress conditions and slightly induced under hypersaline stress conditions (1.15-fold). One possible explanation for this might lie in the fact that transcriptional regulation is not the most important mechanism regulating the activity of these enzymes. This hypothesis would be compatible with an earlier study by Collén and Davison [54], who found that the cellular activity of ROS scavenging enzymes correlated with vertical zonation of different species of the brown algal order Fucales in the intertidal zone. To our knowledge, it is currently not known whether the activity of the ROS scavengers that were examined also changes upon exposure to abiotic stress. Such studies could greatly aid our understanding of the role of these enzymes in the brown algal stress response.

An alternative or additional explanation to that of non-transcriptional mechanisms regulating the activity of ROS scavenging enzymes could be that other, yet unknown proteins and mechanisms play more important roles in the defense against ROS in brown algae. One candidate for this kind of protein could be the chlorophyll a/c binding proteins. Thirty chlorophyll a/c binding proteins were represented on our microarray, most of them being down-regulated mainly under hyposaline and oxidative stress conditions. As chlorophyll a/c binding proteins serve as light-harvesting antennae, this down-regulation is likely to represent a response to the reduced photosynthesis efficiency (quantum yield) under stress conditions. Reducing the amount of energy that reaches the photosynthetic reaction center would also reduce the need for non-photochemical quenching and decrease the risk of the formation of ROS. However, there were also three genes coding for chlorophyll a/c binding proteins among the most significantly up-regulated genes under hyposaline and hypersaline stresses. A similar observation was made by Hwang *et al. *[55] in the Antarctic diatom *Chaetoceros neogracile*, where heat stress induced the up-regulation of five and the down-regulation of ten genes coding for chlorophyll a/c binding proteins. It is possible that these up-regulated chlorophyll a/c binding proteins, in spite of their high sequence similarity with the other transitionally down-regulated ones, have evolved or are evolving to serve different functions within the heterokont lineage.

### Ions and potential osmolytes

In parallel to the induction of 'classical' stress response genes, we observed the transcriptional regulation of several genes involved in the synthesis or degradation of potential organic osmolytes and the transport of ions under saline stress. Under hypersaline stress, changes in the extracellular salt concentration as well as cell volume are likely to cause imbalances in ion concentrations, explaining the need for transporters to maintain homeostasis. Two genes coding for a magnesium and a potassium transporter were up-regulated specifically under hypersaline stress. Interestingly, we did not observe transcriptional activation of sodium transporter genes under salt stress, although many glycophytes (non- or moderately salt tolerant terrestrial plants) use these transporters to exclude NaCl from their cytosol, allowing a certain degree of salt tolerance [56]. The latter observation suggests that the large quantities of NaCl accumulated upon exposure to saline stress (Figure 2) are stored within the cytoplasm rather than in the vacuole. A similar observation was made by Miyama and Tada [57] in the Burma mangrove. In this tree, exposure to sub-lethal NaCl stress did not cause an activation of Na transporters but led to a slow increase of the NaCl concentration in the leaves. One possible explanation for this, as proposed by Miyama and Tada [57], is that NaCl itself could serve as an osmolyte within the cells of the Burma mangrove. This may also be true for brown algae - a hypothesis that is strengthened by the observation that, in *Ectocarpus *as well as in *Lamninaria *[58] and the Burma mangrove [57], sorbitol, added at the same osmolarity as NaCl, had irreversible toxic effects.

While there is no evidence of the synthesis of additional compatible osmolytes in the Burma mangrove, we observed an increasing difference between intracellular osmolarity and intracellular Na^+ ^concentration with rising salinity in *E. siliculosus*, demonstrating the accumulation of other osmotically active substances in the cell. Mannitol has frequently been suggested to be a compatible osmolyte in brown alga [16,17], and indeed, one of the two mannitol 1-phosphate-dehydogenases in *E. siliculosus *was down-regulated in hyposaline stress, and the other up-regulated in hypersaline stress (though with a weak *P*-value of 0.066). In addition, under hyposaline conditions, we observed a strong up-regulation of a proline dehydrogenase gene (Additional data file 3), an enzyme responsible for the degradation of proline, which is known as a compatible osmolyte in higher plants [59] and diatoms [60]. Degrading proline under conditions of low salinity might help *E. siliculosus *to reduce the osmotic pressure between the intracellular and extracellular medium. Finally, a possible role of urea as a compatible osmolyte was suggested in diatoms [61]. *E. siliculosus *possesses the complete urea cycle and genes encoding arginases were up-regulated in hypersaline conditions. As arginases catalyze the last reaction of this cycle - that is, the degradation of arginine to ornithine and urea - their up-regulation supports the hypothesis of urea as a compatible osmolyte in heterokonts. There are, however, other or additional possible roles of arginases: in higher plants, for example, arginases have been shown to play a regulatory role in nitric oxide metabolism, increasing both the synthesis of proline and polyamines [62], both of which, in turn, are part of their osmotic stress response [59]. Additional experiments addressing the question of the possible compatible osmolytes in brown algae - for example, metabolite profiling - are now required to further test these hypotheses.

### Stress response genes with unknown functions

Although our study has revealed several major themes underlying the abiotic stress response of *E. siliculosus*, we should not forget that this analysis was based on only a subset of the genes that actually changed expression. Our manual analysis of the 966 of the most significantly regulated genes has shown that 53.7% of these genes, to date, have no known homologs in current databases, including diatoms, and for another 12.6% there is no indication of their function, even though homologs exist in other organisms. This demonstrates both the discovery potential working with *E. siliculosus *and the amount of work that lies ahead for the phycological community.

## Conclusions

In this study, which presents the first large-scale transcriptomic study within the brown algal lineage, we have developed and compiled the tools and protocols necessary to perform microarray experiments in the emerging model brown alga *E. siliculosus*, and used these tools to study the transcriptional response to three different stresses. Our results show that *E. siliculosus *undergoes large-scale transcriptomic reprogramming during the short-term acclimation to abiotic stress. The observed changes include several modifications to transcription, translation, amino acid metabolism, protein turnover, and photosynthesis, and indicate a shift from primary production to protein and nutrient recycling.

Although *E. siliculosus *shares many stress responses with flowering plants, for example, the induction of genes involved in vesicular trafficking, some classical stress responses, such as the induction of several ROS scavengers, could not be observed. On the other hand, our data highlighted many novel reactions such as the up-regulation of several genes coding for chlorophyll a/c binding proteins or the regulation of a large percentage of unknown genes, many of which are unique to *E. siliculosus*. In particular, the latter result, that is, that the functions of two-thirds of the regulated genes are unknown, underlines the fact that many of the molecular mechanisms underlying the acclimation to environmental stresses in brown algae are still entirely unknown. Understanding these mechanisms is a challenge that will still require much research, and our study provides a valuable starting point to approach this task.

## Materials and methods

### Growth conditions, stress treatments, and measurements of osmolarity and Na^+ ^concentration

*E. siliculosus *(Dillwyn) Lyngbye (Ectocarpales, Phaeophyceae) unialgal strain 32 (accession CCAP 1310/4, origin San Juan de Marcona, Peru) was cultivated in 10-liter plastic flasks in a culture room at 14°C using filtered and autoclaved natural seawater enriched in Provasoli nutrients [63]. Light was provided by Philips daylight fluorescence tubes at a photon flux density of 40 μmol m^-2 ^s^-1 ^for 14 h per day. Cultures were bubbled with filtered (0.22 μm) compressed air to avoid CO_2 _depletion. Ten days before the stress experiments, tissues were transferred to ASW with the following ion composition: 450 mM Na^+^, 532 mM Cl^-^, 10 mM K^+^, 6 mM Ca^2+^, 46 mM Mg^2+^, 16 mM SO_4_^2-^.

Three different stress media were prepared based on ASW. For hyposaline stress, ASW was diluted to 12.5% of its original concentration with distilled water, resulting in a final NaCl concentration of 56 mM. For hypersaline stress, ASW with a final concentration of 1.47 M NaCl was used. For oxidative stress, H_2_O_2 _(30% w/w; Sigma-Aldrich, St. Louis, MO, USA) was added to the ASW immediately before beginning the stress experiment at a final concentration of 1 mM. Identical quantities of Provasoli nutrients were added in each of these media.

In order to monitor the intensity of a stress, we measured the quantum yield, a fluorometric marker for the photosynthetic efficiency, using a Walz Phyto-pulse amplitude modulation fluorometer (Waltz, Effeltrich, Germany) and default parameters (actinic light intensity 3, approximately 90 μE m^-2 ^s^-1^; saturation pulse intensity 10, approximately 2,000 μE m^-2 ^s^-1^, 200 ms) before harvesting the cultures.

The stress experiment was started by filtering 20 liters of ASW-acclimated *E. siliculosus *and transferring approximately 4 g of tissue each to 20 flasks (5 replicates per condition) containing 1 liter of one of the three stress media or the control medium (ASW with Provasoli nutrients). After 6 h the content of each flask was harvested by filtration, dried with a paper towel, and immediately frozen in liquid nitrogen.

Immediately after harvesting, about 300 mg (wet weight) of sample were thoroughly ground at room temperature (RT) and centrifuged for 1 minute at 16,000 g. Both the supernatant and a sample of the culture medium were then used to measure the concentration of osmolytes employing a freezing point depression osmometer (Osmometer Type 15, Löser Messtechnik, Berlin, Germany), and to determine the intracellular concentration of Na^+ ^with a FLM3 flame photometer (Radiometer, Copenhagen, Denmark).

### Sequence preparation and array design

The 90,637 EST sequences used for the microarray design were cleaned using Phred [64] (trim-cutoff 0.05) and SeqClean and assembled using TGICL [65] and default parameters. Forty-one sequences that had been removed by Phred were re-included in the dataset, because they had significant BLAST hits with known eukaryotic proteins. In addition, 231 *E. siliculosus *virus 1 (Es-V1) sequences and 21 genomic intron sequences were included in the design. All assembled sequences are available directly from our homepage [22] and the ESTs have been deposited in public databases [EMBL: FP245546-FP312611]. Relevant accession numbers are listed in Additional data file 8.

Four 60-mere probes were designed for 17,119 of the 17,332 sequences (132 genes are not represented on the array) by Roche NimbleGen (Madison, WI, USA) and synthesized on 4-plex arrays with 72,000 features per hybridization zone. Roche NimbleGen also carried out cDNA labeling and hybridization as part of their gene expression array service.

### Automatic annotation and correspondence table

All sequences were automatically annotated with KEGG orthology (KO) numbers using KOBAS [37] and with GO terms [66] using GOPET [36]. Protein sequences corresponding to the assembled EST sequences were then predicted using ORF predictor [67]. The automatic annotation of these sequences yielded 2,383 and 3,148 annotated sequences, respectively. As 37.5% of the cDNA sequences that were represented on the array contained mainly, or exclusively, 3' untranslated region sequence, their function could not be assessed directly. In these cases the corresponding genome sequence was annotated, yielding an additional 1,047 KO and 2,743 GO annotations. The correspondence table used to relate the assembled ESTs to supercontigs was generated by blasting all of the assembled EST sequences against the full *E. siliculosus *genome (coding and non-coding sequences) and selecting the best hit (best identity, longest alignments). Wherever this best hit was part of a predicted coding sequence (CDS), the corresponding CDS was chosen. In cases where the hit region was upstream of only one CDS, this CDS was chosen. In some cases the best hit was located upstream of two CDSs on opposite strands. Here the closest CDS was selected, if the distance to the closer CDS was half as long as or shorter than that to the other CDS. Otherwise no corresponding genome sequence was selected. In total, 3,430 (20%) of all represented genes were annotated with KEGG and 5,891 (34%) were annotated with GO terms.

### Sample preparation, hybridization and verification

RNA was extracted from approximately 100 mg (wet weight) of tissue following Apt *et al. *[68] with modifications as described by Le Bail *et al. *[28], using a cetyltrimethylammonium bromide (CTAB)-based buffer and subsequent phenol-chloroform purification, LiCl-precipitation, and DNAse (Turbo DNAse, Ambion, Austin, TX, USA) steps. RNA quality and quantity was then verified on 1.5% agarose gel stained with ethidium bromide and a NanoDrop ND-1000 spectrophotometer (NanoDrop products, Wilmington, DE, USA). For four of the five flasks, mRNA was isolated from the total RNA using the PolyATtract^® ^mRNA Isolation System III (Promega, Madison, WI, USA). These samples were concentrated in a SpeedVac concentrator (Savant, Ramsy, MN, USA) and again quantified using the NanoDrop. One replicate was used to verify if our procedure would directly work from total RNA.

Double-stranded cDNA was synthesized and amplified with the SMART cDNA synthesis kit (Clontech, Mountain View, CA, USA) and the M-MuLV reverse transcriptase (Finnzymes, Espoo, Finland) starting from 30 ng of mRNA or 100 ng of total RNA. In this kit, the first strand of cDNA is synthesized using an oligo(dT) primer with an attached SMART priming site. The terminal C-transferase activity of the reverse transcriptase will create an oligo(dC) tail at the 5' end of each mRNA, which is used to add a second SMART priming site. The two priming sites are then used to produce the second strand of the cDNA and to amplify it by PCR according to the Clontech protocol, using the Advantage2 polymerase (Clontech). The optimal number of amplification cycles was determined by semi-quantitative PCR, and ranged between 20 and 25 for our samples.

The PCR reactions were purified by first vortexing with one volume of phenol:chloroform:isoamyl alcohol (25:24:1), then by precipitating the aqueous phase with 0.5 volume of 7.5 M NH_4_OH, 6 μg of nuclease-free Glycogen (Ambion), and 2.4 volumes of ethanol. After centrifugation at RT (20 minutes at 14,000 g), the pellet was washed with 70% EtOH, centrifuged (10 minutes at 14,000 g and RT) and resuspended in 14 μl of H_2_O. To finish, cDNAs were once more quantified and checked on an agarose gel to ensure that they met the requirements of Roche NimbleGen for hybridization (concentration >250 μg l^-1^, A_260/280 _≥ 1.7, A_260/230 _≥ 1.5, median size ≥ 400 bp).

RT-qPCR validation of the microarray was performed as described by Le Bail *et al. *[28].

### Statistical analysis

Expression values were generated by Roche NimbleGen using quantile normalization [69], and the Robust Multichip Average algorithm [70,71].

To increase the power of subsequent statistical tests, non-expressed genes were removed from the dataset. This was done by comparing the raw expression values of each gene with those of the random probes included in the array design by Roche NimbleGen. Most of the random probes (>99.66%) had raw expression values inferior to 450, so all genes that had raw expression values over 450 in one of the replicates of the different experimental conditions were considered expressed. The probability p' of labeling a non-expressed gene as being expressed can be calculated according to the laws of a binomial distribution. Since 16 hybridizations were considered, for our dataset p' equals 5.3%, meaning that we have theoretically removed 94.7% of all genes without detectable expression.

The most stable genes were defined by ranking the genes according to the sum of squares of the log2-ratios of all stress conditions with the control. Differentially expressed genes were identified in the reduced dataset by *t*-test using TigrMEV 4.1 [72] with subsequent calculation of the FDR according to Benjamini and Hochberg [73]. Clustering was performed on the log2-ratios of all expressed genes with the control, considering all mRNA replicates using TigrMEV 4.1. A k-means algorithm [74], 'Euclidian distance', and 'average linkage' were selected. The ideal number of clusters for our dataset (k = 7) was determined using a figure of merit graph [75]. After clustering, the different replicates were averaged to generate the expression graphs. Both the clusters and the over-expressed and repressed genes identified by the *t*-test (FDR 10%) were analyzed to identify over-represented groups of genes. Over-expressed KEGG categories were identified using the KOBAS web-site [37] and a binomial test. Over-represented GO terms were identified using the GO Local Exploration Map (GOLEM) software (version 2.1) [76] and the Benjamini and Yekutieli algorithm to determine the FDR [77]. Additionally, the 966 genes that showed the most significant changes in expression (that is, genes that meet both criteria: significance at an FDR <1% and a relative change in expression compared to the control of more than twofold) were annotated and grouped manually.

### Stress response genes with unknown functions

Genes were considered unknown stress response genes if they significantly changed expression in at least one stress treatment compared to the control (FDR <1%, more than twofold change compared to the control) and when, for both the assembled EST sequence itself and for the corresponding genome sequence, no BLAST hits were found in either the NCBI databases or among the known heterokont genomes (*Phytophthora sojae*, *P. ramosum*, *Thalassiosira pseudonana*, and *Phaeodactylum tricornutum*). Where homologs (e-value < 1e-10) were found, but these homologs had no functional annotations, genes were considered conserved unknown stress response genes. To group these genes, all unknown stress response genes and conserved unknown stress response genes were blasted against themselves using the NCBI BLAST program (version 2.2.18) [78] and a cut-off e-value of 1e-10. If there were several alignments between two genes, only the alignment with the lowest e-value was considered. Self-hits were removed. All groups of genes with homologs among the (conserved) unknown stress response genes in *E. siliculosus *were then visualized using Cytoscape 2.6.0 [79]. Their subcellular localizations were identified using HECTAR [80] and transmembrane domains were searched for using TMAP [81].

### Data deposition

Microarray data have been deposited in a public database [ArrayExpress:E-TABM-578].

## Abbreviations

ASW: artificial sea water; CDS: coding sequence; EST: expressed sequence tag; FDR: false discovery rate; GO: Gene Ontology; GOPET: GO-term Prediction and Evaluation Tool; HSP: heat shock protein; KEGG: Kyoto Encyclopedia of Genes and Genomes; KOBAS: KEGG Orthology-Based Annotation System; NCBI: National Center for Biotechnology Information; ROS: reactive oxygen species; RT: room temperature; RT-qPCR: real time quantitative PCR; SNARE: soluble N-ethylmaleimide-sensitive factor attachment receptor.

## Authors' contributions

SMD, together with and under supervision of TT, performed the experiments, analyzed the data and wrote the manuscript, with help from CB and JMC; DS, BS and JLP have generated cDNA libraries (normalized and non-normalized); CDS was involved in EST data treatment and data formatting for sequence submissions; EC helped assemble the ESTs and set up the internet site; RK and KHG provided the GOPET annotations; MD modified ArrayLIMS and EMMA to work with the Roche NimbleGen arrays; JMC, PR, YVDP, and LS were involved in genome-wide gene annotation; all authors read and approved the manuscript.

## Additional data files

The following additional data are available with the online version of this paper: a figure showing the recovery of the photosynthetic efficiency (quantum yield) in *E. siliculosus *cultures after 24 hours of stress treatment (Additional data file 1); a table listing the KEGG pathways and GO terms identified to be over-represented among the transcripts significantly different in the mRNA and total-RNA-derived samples (Additional data file 2); a table listing the 966 most regulated (FDR <0.01) and the 100 most stable genes on the microarray in all stress conditions (Additional data file 3); a table listing the KEGG pathways identified to be over-represented among the transcripts significantly up- or down-regulated in the different stress conditions (Additional data file 4); a table listing the GO categories identified to be over-represented in the different conditions at a FDR of 10% (Additional data file 5); description of the unknown stress response genes identified in this study (Additional data file 6); a table listing the C5-epimerase-genes represented on the microarray and their expression profiles (Additional data file 7); accession numbers of all sequences used in the design of the *E. siliculosus *microarray (Additional data file 8).

## Supplementary Material

Additional data file 1 The tested stress conditions correspond to the conditions used for the microarray experiment (1,470 mM NaCl, 12.5% seawater, and 1 mM H_2_O_2_), but the duration of the stress treatments was 24 hours rather than 6 hours. Click here for file

Additional data file 2 P indicates the probability of this pathway being actually over-represented in the two datasets (5% FDR and 10% FDR). The column FDR refers to the FDR that was applied when identifying the significantly regulated transcripts used for this analysis. Click here for file

Additional data file 3 All genes are grouped according to their putative function. Click here for file

Additional data file 4 Q represents the q-value, that is, the expected proportion of false positives incurred when the pathway is considered significant. It is given for the two datasets containing genes considered to be significantly regulated at FDRs of 5% and 10%. Pathways significant at q-values < 0.05 are highlighted in boldface; pathways not known to be present in *E. siliculosus *and thus representing possible artifacts of the automatic annotation are shown in grey. Click here for file

Additional data file 5 The column FDR refers to the FDR that was applied when identifying the significantly regulated transcripts used for this analysis. Click here for file

Additional data file 6 The first tab contains a graphical representation of identified groups of unknown stress response genes. The roman numerals on the left (I-VI) refer to the network graphs to their right, each representing a group of unknown stress response genes. In the network graphs, brown nodes represent sequences only found in *E. siliculosus*, while green nodes represent genes found in other organisms also. Edges represent the sequence similarity between two genes (e-value of the BLAST output). The cluster each gene was assigned to by the k-means clustering (Figure 5) is given in boldface. Furthermore, the expression profile of each gene in the three stress conditions (hyposaline-, hypersaline-, and oxidative stress) is given on the right. Yellow indicates that a gene was over-expressed in a particular condition, while black indicates no change, and blue indicates that a particular gene was repressed. The other two tabs contain additional information, such as corresponding genome equivalent, amino acid sequence, subcellular localization of each protein, the results of the transmembrane domain search, and the expression profile for both the genes shown in the first tab and the other stress response genes that could not be assigned to the different groups. Click here for file

Additional data file 7 C5-epimerase-genes represented on the microarray and their expression profiles. Click here for file

Additional data file 8 Accession numbers of all sequences used in the design of the *Ectocarpus siliculosus *microarray. Click here for file
